# Epigenetic and Cellular Reprogramming of Doxorubicin-Resistant MCF-7 Cells Treated with Curcumin

**DOI:** 10.3390/ijms252413416

**Published:** 2024-12-14

**Authors:** Paola Poma, Salvatrice Rigogliuso, Manuela Labbozzetta, Aldo Nicosia, Salvatore Costa, Maria Antonietta Ragusa, Monica Notarbartolo

**Affiliations:** 1 Department of Biological Chemical and Pharmaceutical Science and Technology (STEBICEF), University of Palermo, 90128 Palermo, Italy; paola.poma@unipa.it (P.P.); salvatrice.rigogliuso@unipa.it (S.R.); manuela.labbozzetta@unipa.it (M.L.); salvatore.costa@unipa.it (S.C.); monica.notarbartolo@unipa.it (M.N.); 2 Institute for Biomedical Research and Innovation—National Research Council (IRIB-CNR), 90146 Palermo, Italy; aldo.nicosia@irib.cnr.it

**Keywords:** multidrug resistance, P-glycoprotein, curcumin, breast cancer, DNA methylation, ribosome biogenesis, translation, cytoskeletal dynamics

## Abstract

The MCF-7R breast cancer cell line, developed by treating the parental MCF-7 cells with increasing doses of doxorubicin, serves as a model for studying acquired multidrug resistance (MDR). MDR is a major challenge in cancer therapy, often driven by overexpression of the efflux pump P-glycoprotein (P-gp) and epigenetic modifications. While many P-gp inhibitors show promise in vitro, their nonspecific effects on the efflux pump limit in vivo application. Curcumin, a natural compound with pleiotropic action, is a nontoxic P-gp inhibitor capable of modulating multiple pathways. To explore curcumin’s molecular effects on MCF-7R cells, we analyzed the expression of genes involved in DNA methylation and transcription regulation, including *ABCB1/MDR1*. Reduced representation bisulfite sequencing further unveiled key epigenetic changes induced by curcumin. Our findings indicate that curcumin treatment not only modulates critical cellular processes, such as ribosome biogenesis and cytoskeletal dynamics, but also reverses the resistant phenotype, toward that of sensitive cells. This study highlights curcumin’s potential as an adjuvant therapy to overcome chemoresistance, offering new avenues for pharmacological strategies targeting epigenetic regulation to re-sensitize resistant cancer cells.

## 1. Introduction

Multidrug resistance (MDR) is a phenomenon where cancer cells develop resistance to multiple chemotherapeutic agents, often through mechanisms like enhanced drug efflux, alterations in drug targets, enhanced DNA repair, and evasion of apoptosis pathways. The onset of MDR poses significant challenges to effective cancer treatment, making it a critical area of research in oncology today, despite the development of targeted antitumor chemotherapeutics.

The emerging mechanism of drug resistance depends on the cell type, the cell state, the background set, and the evolving mutational load. Epigenetic dynamics and chromatin organization are key factors that alter the transcriptomes in drug-resistant clones. It is known that modification in DNA methylation by DNMTs (DNA methyltransferases) and TET (ten-eleven translocation) enzymes can result in transcriptional silence of tumor-suppressive genes and transcriptional activation of proto-oncogenes and that treatments of MDR cells with DNA methyltransferase inhibitors can decrease the chemoresistance [1]. Several studies were performed using MCF-7 cells, an epithelial cancer cell line derived from breast adenocarcinoma, as a cellular model. For example, it was found that the promoter region of genes with a significant role in resistance, such as *ABCB1* (*MDR1*, encoding the ATP-dependent translocase ABCB1 or P-glycoprotein), are significantly differentially methylated in MCF-7 and in drug-resistant MCF-7 cells [2,3,4,5].

In particular, multidrug resistance acquired after pharmacological treatment, due often to the overexpression of drug transporters such as P-glycoprotein (P-gp), is a challenge to be faced.

Therapeutic interventions to counteract the MDR phenomenon focus on the use of transporter inhibitors, which can also be used in combination with antitumor chemotherapy drugs.

We and other research groups have demonstrated how natural substances identified as “fourth-generation P-glycoprotein inhibitors” have a potential use in combination with antitumor chemotherapeutics [6,7,8]. The advantage of using natural molecules lies in their lack of toxicity and their ability to act on the overexpression of P-gp at various levels and through different mechanisms of action. This group of molecules certainly includes curcumin, a polyphenol extracted from the rhizomes of *Curcuma longa*, which can modulate both the function and expression of P-gp [9]. In addition to inhibiting P-gp, both functionally and in expression, curcumin is a multitarget substance with a mechanism of action that affects numerous altered signaling pathways in the neoplastic cell. It can influence MDR through epigenetic regulation and modulation of various transcription factors, as well as oncoproteins, apoptosis inhibitory factors, growth factors, and protein kinases. Moreover, numerous studies have highlighted that curcumin is able to reverse drug resistance by activating various cellular signaling pathways also through epigenetic regulation [10,11,12,13].

Based on these considerations, in the present study, we used the doxorubicin-resistant variant of MCF-7 cells (MCF-7R) to determine if curcumin can revert MDR phenotype through the reversal of the DNA methylation aberrations associated with the acquisition of doxorubicin resistance.

## 2. Results and Discussion

### 2.1. Effects of Curcumin on P-Glycoprotein Expression

The MCF-7R cellular model utilized in this study derives from the parental sensitive MCF-7 cell through repeated exposure to gradually increasing concentrations of doxorubicin. This treatment causes constitutive activation of the NF-κB transcription factor, an overexpression of P-gp and other typical factors related to apoptosis inhibition [13,14,15].

Curcumin exhibits an IC_50_ value of 30 µM on the MCF-7R cell line after 72 h of treatment (Supplementary Material Figure S1). The same concentration after 24 h determines a subcytotoxic effect (80% viability), and for this reason, it was used for subsequent molecular analyses. Furthermore, curcumin is nontoxic at concentrations up to 50 µM after 24 h of treatment in a nontumorigenic cell line (human mammary epithelial cells, HMEpiC; Supplementary Material Figure S2).

Herein, we wonder to evaluate the action of curcumin on the expression of P-gp in MCF-7R cells.

**Figure 1:**
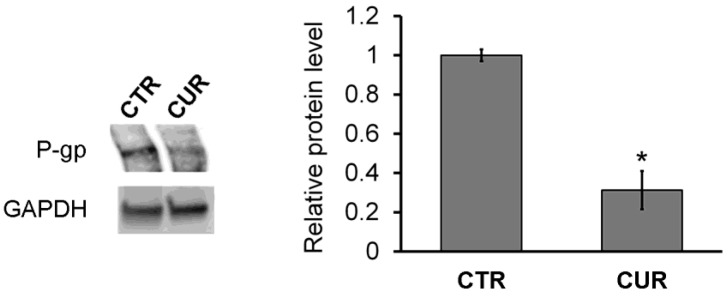
Western blotting analysis of P-gp levels. Cells were treated with curcumin (30 µM) for 24 h. “CTR” refers to the MCF-7 doxorubicin-resistant cells and “CUR” refers to the MCF-7 doxorubicin-resistant cells treated with curcumin for 24 h. The results are expressed as the mean ± standard error of two different experiments. Differences when treatment is compared to control: * *p* < 0.05 (one-way ANOVA followed by Tukey’s test).

The treatment with curcumin (30 µM) for 24 h produced a strong reduction in the P-gp protein level, observed by Western blotting (Figure 1). Curcumin can reduce the activation of NF-κB of which the P-gp efflux pump is a target. This action has been widely demonstrated in various tumor types and confirmed by our analysis via TransAM assays [15,16,17]. By measuring the DNA-binding capacity of NF-κB (p65 subunit) in the nuclear extracts of MCF-7R cells before and after treatment with 30 µM curcumin, we observed a 10-fold inhibition of NF-κB activation after 24 h (Table 1). Furthermore, we have previously reported no changes in the levels of NF-κB activation in the same cell model, MCF-7R, following very short treatments with curcumin (4 and 8 h) [18]. This suggests a time-dependent effect of curcumin on MCF-7R cells.

**Figure 2:**
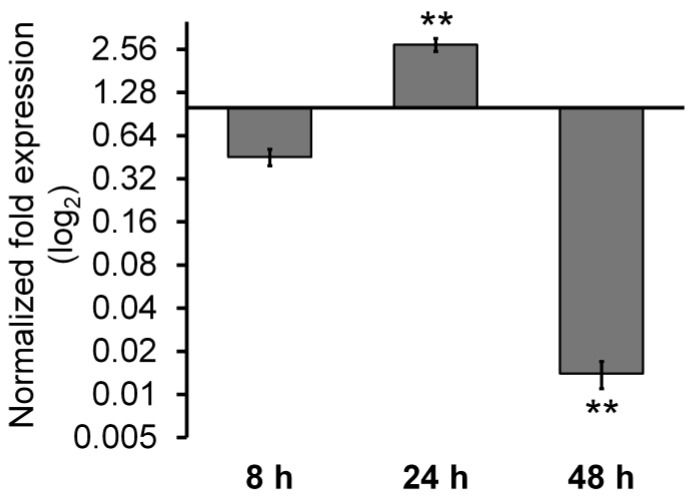
Evaluation of P-gp mRNA expression levels by qRT-PCR. For each condition, N = 3 technical replicates were used. Data are expressed as mean ± standard error of two experiments. Cells were treated with curcumin (30 µM) at the indicated time. ** (*p* < 0.01) represent significant differences among the times (one-way ANOVA followed by Tukey’s test).

The same trend in *P-gp* mRNA levels would be expected. Instead, we observe a fluctuating action of curcumin on the transcript level in a time-dependent way (Figure 2). Curcumin exposure (30 µM) reduces *P-gp* mRNA at 8 h, then causes an increase at 24 h and a strong reduction at 48 h. Considering the P-gp expression profile, the activity of NF-κB and the relative mRNA level, it is reasonable to hypothesize that different layers of regulation, including transcriptional and post-transcriptional mechanisms, act to tune the expression level of P-gp in the presence of curcumin.

### 2.2. Effects of Curcumin on the MCF-7R DNA Methylation Landscape

#### 2.2.1. Global Analysis of RRBS Data

It has been reported that curcumin can reduce the activities of DNMTs, thus resulting in changes in the DNA methylation rate. However, this activity could be cell type or tissue-specific because no significant global DNA hypo-methylation was observed in both leukemia and colorectal cancer in response to curcumin treatments [11].

Therefore, we tested curcumin’s effects in our experimental setting.

**Figure 3:**
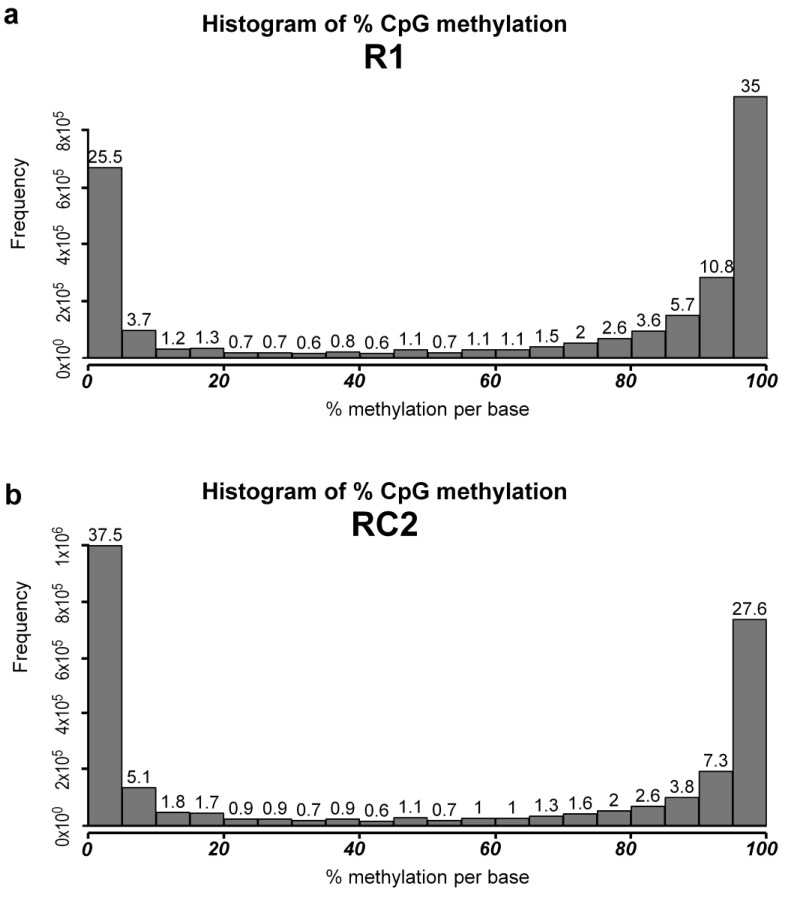
Reduced representation bisulfite sequencing (RRBS) results. Statistical analysis of global data of a representative MCF-7R untreated cell line (R1, (**a**)) and an MCF-7R sample treated with curcumin (RC2, (**b**)).

In order to evaluate if curcumin (30 µM for 24 h) modulates the DNA methylation landscape of MCF-7R cells, reduced representation bisulfite sequencing (RRBS) analyses were carried out. RRBS data statistical analysis shows that cell treatment with curcumin has an overall demethylating effect. Untreated MCF-7R cells showed about 25% demethylated CpG sites and about 35% strongly methylated sites. Differently, MCF-7R curcumin exposure provided about 37% demethylated CpG sites and about 28% strongly methylated sites (Figure 3).

Correlation analysis shows a very high correlation index (0.97%), indicating a strong direct relationship of data; therefore, the curcumin effect is specific for particular regions.

RRBS data were analyzed not only by selecting differently methylated CpGs (DMCs) but also by selecting differently methylated “tiles” (200 bp and 1000 bp tiles). To highlight the significant variations in DNA methylation, all found DMCs were aggregated in regions 200 bp long. All aggregated DMCs and tiles with a |methylation difference| > 25% at q-value < 0.01 were considered significant (differently methylated regions, DMRs). All data are reported in the Supplementary Material Table S1. The demethylating effect of curcumin is confirmed by the observation that 83% of DMRs are less methylated.

Also analyzing the differences between MCF-7R and MCF-7 parental sensible cells, results indicate that at least 64% of demethylated DMRs after the treatment with curcumin (with significant meth. diff value also in MCF-7R-S) are hypermethylated with the acquisition of resistance to doxorubicin.

**Figure 4:**
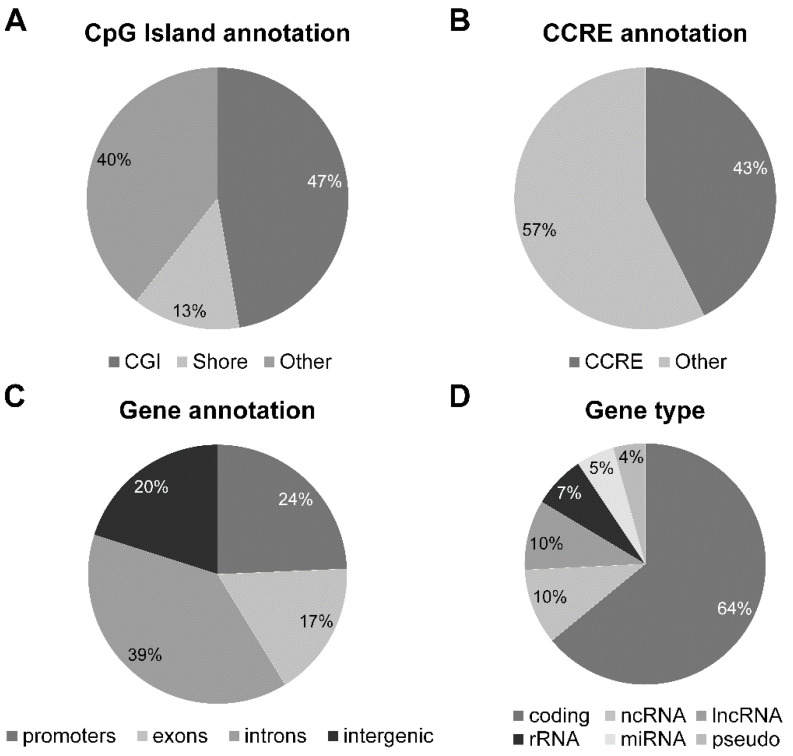
Annotations of all DMRs (aggregated DMCs and tiles). (**A**) CpG island annotation, (**B**) ENCODE candidate Cis-Regulatory Elements annotation, (**C**) gene annotation, (**D**) gene class annotation.

The DMRs were annotated with respect to CpG islands (CGI and shores), ENCODE candidate Cis-Regulatory Elements (cCREs), and genes (Figure 4A–C). The differentially methylated gene list was sorted with respect to the gene type (Figure 4D).

Then, all differently methylated promoters or cCRE (n = 257) were considered for a gene ontology enrichment analysis. Genes were grouped by functional categories defined by high-level GO Biological Process terms. The most highly enriched gene ontology terms were Anatomical structure morphogenesis (GO:0009653), Response to chemical (GO:0042221), and Positive regulation of metabolic process (GO:0009893).

**Figure 5:**
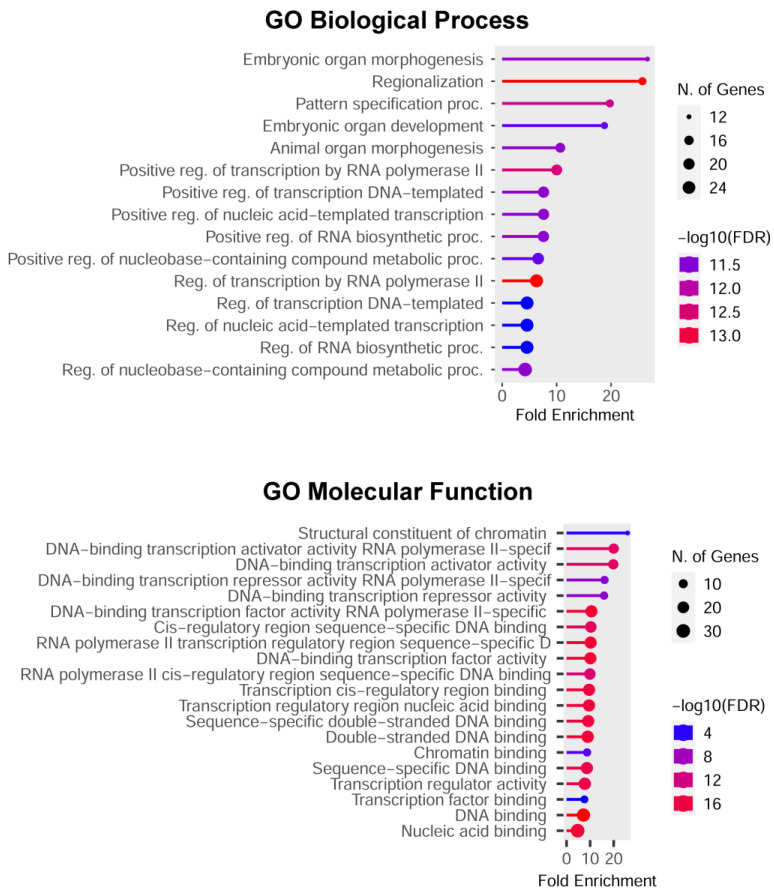
GO enrichment analysis performed on 33 differentially methylated genes belonging to Chromatin or Chromosome GO terms.

Thirteen percent of the items are grouped in Chromatin and Chromosome GO Cellular Component terms. As shown in Figure 5, they include TFs regulating morphogenesis, development, and pattern specification, via RNA biosynthesis regulation.

#### 2.2.2. Curcumin Affects the rRNA Loci Methylation Rate

**Figure 6:**
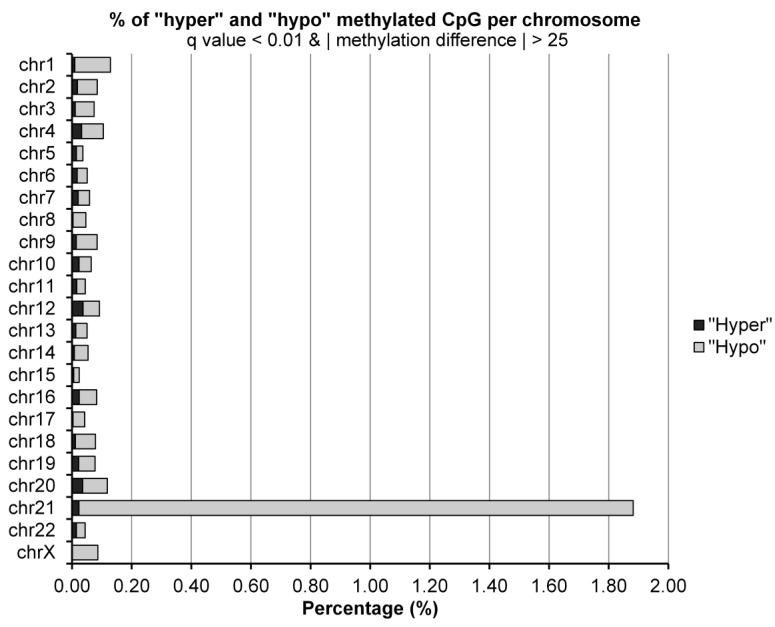
Distribution of differentially methylated CpGs per chromosome.

Because the distribution of differentially methylated cytosines (DMCs) across chromosomes is crucial in studying the epigenetic landscape, efforts were made to define chromosomal distribution patterns of DMCs modifications in MCF-7R in response to curcumin exposure. The distribution of DMCs on different chromosomes shows a surprisingly high percentage of demethylated CpG in chromosome 21 (1.86%), with respect to other chromosomes (0.13%—Figure 6).

**Figure 7:**
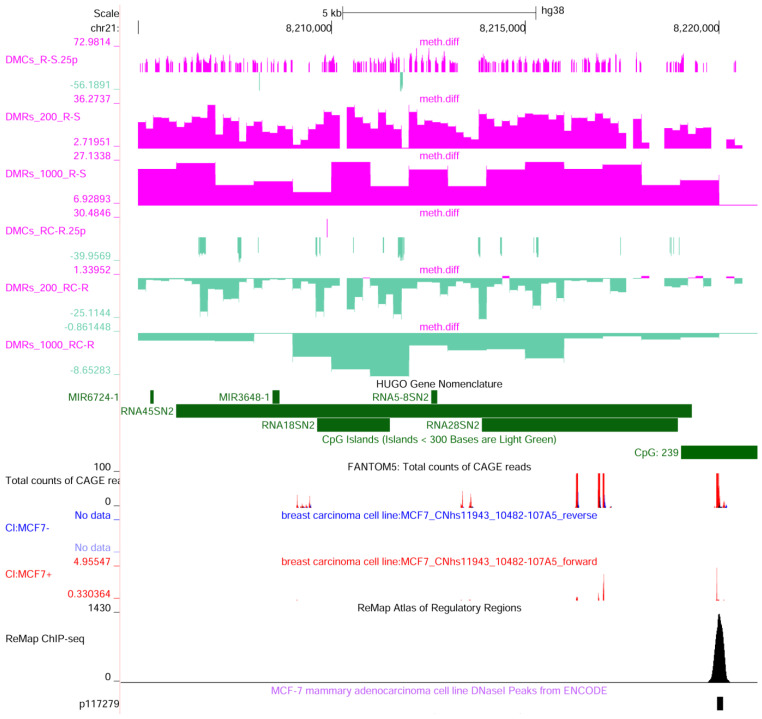
UCSC genome browser view of the 45S ribosomal DNA. This gene represents a copy of the 45S ribosomal RNA on chromosome 21 (chr21:8,204,556–8,220,997). The 45S rDNA repeat unit encodes a 45S rRNA precursor, transcribed by RNA polymerase I, which is processed to form the 18S, 5.8S and 28S rRNAs. Under the chromosome scale, the following are shown: RRBS data (methylation difference R-S: significant DMCs, 200 bp tiles and 100 bp tiles; methylation difference RC-R: significant DMCs, 200 bp tiles and 100 bp tiles), HUGO gene annotation, CGI, Fantom5 CAGE peaks (mapped TSS: total counts in several cell types and MCF-7 data—forward in red and reverse in blue), ReMap Atlas of Regulatory Regions filtered by MCF-7, and DNAseI hypersensitivity in MCF-7.

**Figure 8:**
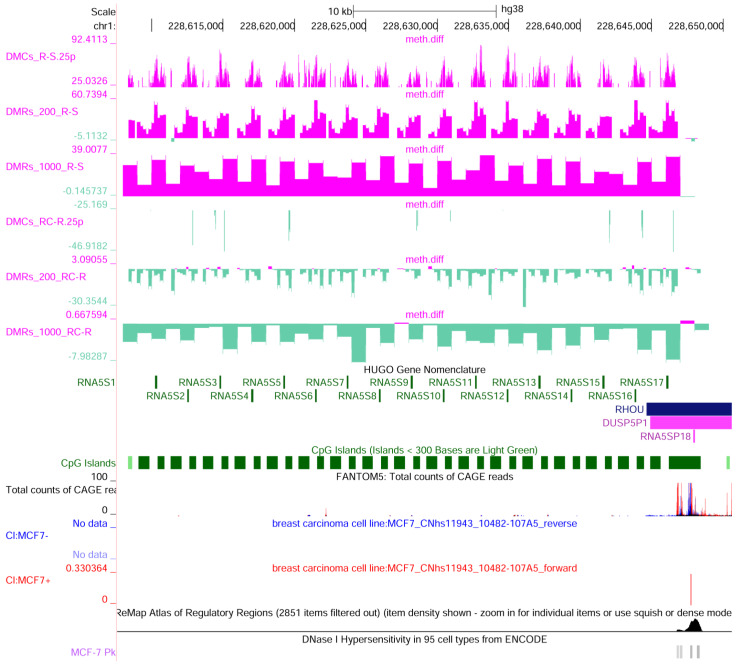
UCSC genome browser view of the 5S ribosomal DNA locus. In this region, there are 17 copies of the 5S ribosomal RNA (chr1:228,607,600–228,650,600). The 5S rDNA is transcribed by RNA polymerase III. On the right, the upstream TSS for RHOU is visible. Under the chromosome scale, the following are shown: RRBS data (methylation difference R-S: significant DMCs, 200 bp tiles and 100 bp tiles; methylation difference RC-R: significant DMCs, 200 bp tiles and 100 bp tiles), HUGO gene annotation, CGI, ENCODE cCRE, Fantom5 CAGE peaks (mapped TSS: total counts in several cell types and MCF-7 data—forward in red and reverse in blue), ReMap Atlas of Regulatory Regions complete and filtered by MCF-7, and DNAseI hypersensitivity in MCF-7.

To assess if a specific distribution of DMCs occurs in the chromosome, an inspection of their localization was carried out. As a result, the differently methylated region mainly maps on chr21:8,204,001–8,475,000. This region corresponds to the repetitive 45S ribosomal DNA (rDNA) arrays encoding the precursor of the principal ribosomal RNAs (Figure 7). Interestingly, the repetitive region for the 5S ribosomal RNAs (*RN5S1*-*RNA5S17*—chr1:228,607,600–228,648,600) on chromosome 1 shows a similar profile (Figure 8). Among other demethylated regions, mapping on different chromosome bands, NOC2-like nucleolar associated transcriptional repressor (*NOC2L*), ribosomal RNA processing 15 homolog (*RRP15*), and DEAD-box helicase 56 (*DDX56*) are also involved in ribosomal biogenesis. All these loci are significantly methylated in MCF-7R and demethylated after the treatment with curcumin.

In breast cancer cell lines, an unexpected hypermethylation has been observed at the promoter region of the *45S* rRNA genes [19]. It has been reported that demethylation of these genes does not significantly reduce the binding phase of Pol I to the rDNA promoter, but there is a slowdown in the subsequent elongation phase. It was suggested that the loss of methylation could induce the production of alternative transcripts from cryptic promoters, potentially by RNA polymerase II, which may be “competing” with Pol I for access to these regions [20]. From the observations presented in Figure 7, CAGE analysis specific to MCF-7 cells reveals the presence of transcription start sites for Pol II within the *RNA45S* gene and near regions showing hypermethylation in the S-R analysis, where methylation trends reverse following curcumin treatment. Consequently, this hypomethylation may enable Pol II to compete with Pol I and initiate transcripts from cryptic promoters. This effect may also result in these transcripts interfering with *RNA45S* maturation, leading to an accumulation of unprocessed rRNA. Such accumulation could potentially drive cells into apoptosis through activation of the nucleolar stress response pathway. In this process, *5S rRNA* plays a central role, forming a trimeric complex with ribosomal proteins RPL5 and RPL11 that directly binds MDM2. This binding prevents MDM2 from inhibiting p53, leading to p53 accumulation, cell cycle arrest, and potentially apoptosis [21].

Thus, it is plausible that curcumin, by negatively affecting the processing of pre-mRNA 45S and positively influencing 5S synthesis, could induce a state of nucleolar stress in tumor cells. In addition, alterations in the assembly of the 60/65S preribosomal particles, possibly due to modifications in protein levels required for ribosome maturation [22,23,24], could interfere with metabolic processes and proliferation. This may prevent the tumor cell from sustaining the protein overproduction required for its survival while simultaneously activating the nucleolar stress response mechanism, leading to cell cycle arrest and apoptosis.

**Figure 9:**
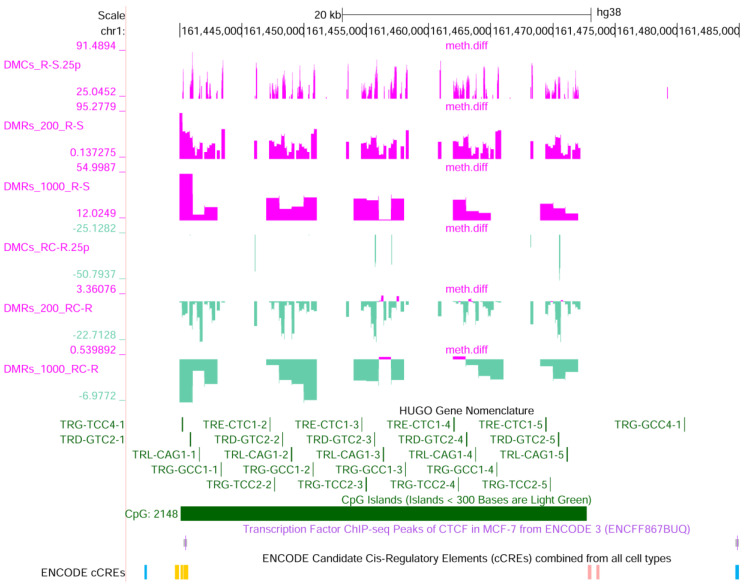
UCSC genome browser view of the differentially methylated CGI on chromosome 1 containing 23 tRNA genes (chr1: 161,440,000–161,472,000). Transfer RNA precursors are transcribed by RNA polymerase III. Under the chromosome scale, the following are shown: RRBS data (methylation difference R-S: significant DMCs, 200 bp tiles and 100 bp tiles; methylation difference RC-R: significant DMCs, 200 bp tiles and 100 bp tiles), HUGO gene annotation, CGI, CTCF binding sites in MCF-7, and cCRE (CTCF-only ENCODE Classification is blue).

Remarkably, the large tandem repeat that displays a high GC content in chromosome 1 (q23.3), each repeat unit containing five tRNA genes (tRNA-Gly-TCC, tRNA-Gly-GCC, tRNA-Asp-GTC, tRNA-Leu-CAG, tRNA-Glu-CTC), is also methylated in MCF-7R and demethylated after exposure to curcumin (chr1: 161,440,000–161,472,000) (Figure 9). This locus shows extensive copy number variation with 9–43 repeat units per allele, displays evidence of meiotic and mitotic instability, and could act as a boundary element also because of the presence of CTCF binding sites [25]. It has been shown that the level of tRNAs may variate across different conditions; indeed, in tumors versus normal breast tissues, both nuclear- and mitochondrial-encoded tRNAs increase up to 10-fold [26]. The peculiarity of the DNA methylation in this region, with respect to the nearby loci, together with the location of CTCF binding sites, suggest an independent regulation of chromatin dynamics and accessibility.

#### 2.2.3. Curcumin Affects Cytoskeletal Dynamics, WNT Pathway, and Transcriptional and Epigenetic Regulation

To highlight the most significant curcumin effects, DMRs containing at least four DMCs in enhancers or promoters that also showed a reversion of the DNA methylation rate compared to control MDR-7 cells were selected. Among the differentially methylated genes, a subset of these is related to cytoskeletal dynamics, by influencing the structural components (like actin filaments and microtubules) and by modulating signaling pathways (e.g., Rho and Rac GTPases) that control cytoskeletal arrangement and cellular movement. A tile in the *RHOU* gene (encoding RhoU, a small GTPase) was one of the most hypermethylated in MCF-7R cells. It is worth noting that *RHOU* is located near the rRNA *5S* repetitive locus and could be influenced by the regulation of ribosomal RNA transcription (Figure 7). Demethylated genes are also CDC42 binding protein kinase gamma (*CDC42BPG*) that may act as a downstream effector of CDC42 (a member of the Rho GTPase family); an isogene of beta-tubulin (*TUBB2B*); syntrophin beta 1 (*SNTB1*); chimerin 2 (*CHN2*, a GAP of the small GTPase Rac1); phosphatidylinositol-3,4,5-trisphosphate dependent Rac exchange factor 2 (*PREX2*, a GEF of Rac1); potassium channel genes (*KCNH6* and *KCNQ4*) that interact with cytoskeletal elements; and *POGZ* that also influences microtubule dynamics and cell division processes. Interestingly, *RAP1GAP2* is also differentially methylated. The *RAP1GAP2* gene encodes a GAP of the small GTPase Rap1, a regulator of cell adhesions and junctions, cellular migration, and polarization that plays many roles during cell invasion and metastasis in different cancers [27]. These results confirm the activity of curcumin in the alteration of cytoskeletal organization and cell motility also in MCF-7R MDR tumor cells [28].

It is known that the Wnt/β-catenin pathway regulating cellular proliferation, migration, and differentiation is usually involved in MDR; interestingly, exposure to curcumin led to the demethylation of several genes within this pathway. Among them, the most significant DMRs mapped on genes belonging either to canonical (*WNT3*, *WNT9B*, and *RSPO3*) and noncanonical Wnt signaling pathways (*PRICKLE2*), thus also involving cell polarity signaling crucial for the organization of cell layers. Furthermore, differentially methylated genes include Wnt/β-catenin–dependent transcriptional factors. These include *NKX2-5*, *HOXB13*, and *HOXD*1, which are master regulators of proliferative/differentiation switches or regulators of the Wnt pathway activity, like *MSX*, which is known to induce the expression of four different Wnt pathway inhibitor genes [29]. Interestingly, in many tumors, the level of Wnt/β-catenin activity is positively correlated with resistance to various chemotherapeutic drugs. Furthermore, chemotherapy treatment can increase Wnt signaling, and this increase confers to the cell the ability to enhance DNA damage repair, facilitating transcriptional plasticity and promoting immune evasion [30].

Curcumin exposure also resulted in changes in the methylation state of epigenetic regulators. As reported in Table 2, several genes displaying differential methylation, such as *MEN1, CBX2, CBX4, CBX8, RING1, NOCL2*, and *MBD3L1*, are integral parts of complexes that play essential roles in regulating epigenetic mechanisms. MEN1 is an essential component of an MLL/SET1 histone methyltransferase (HMT) complex, acting as H3K4 methylase and serving as a tumor suppressor. CBX and RING1 are components of the Polycomb repressive complex 1 (PRC1), which mediates monoubiquitination of histone H2A “Lys-119” acting with PRC2 to silence many genes, including Hox genes. MBD3L1 has a role in DNA methylation, impacting gene expression patterns and potentially influencing cancer development through epigenetic modulation.

Notably, demethylation was observed in the H3 histone gene (*H3C8*) and the *H1-0* gene, which encodes the most abundant variant at nucleoli-associated DNA domains of the linker histone, H1 [31]. Additionally, the product of *POGZ* promotes chromatin accessibility, destabilizing the HP1-chromatin interaction and enabling the activation of Aurora kinase B, which is crucial for mitotic progression. Interestingly, the gene for Aurora kinase C (*AURKC*), which can phosphorylate histone H3 in vitro and is part of the chromosomal passenger complex along with Aurora B, also shows curcumin-dependent demethylation [32]. Additionally, *NOC2L*, besides being a regulator of ribosome biogenesis in the nucleolus, is also an inhibitor of histone acetyltransferase activity that interacts with Aurora B. Aurora B, NOC2L, and P53 form a complex in which NOC2L bridges Aurora B to phosphorylate P53 mainly within the DBD, leading to the repression of P53 function [22].

The hypomethylating activity of curcumin has been ascribed to DNMTs inhibition [11]. Herein we show that such demethylation is not uniformly distributed on the genome, but it is specifically carried out on selected DNA genes/loci. Because some of them encode important chromatin regulators, we can suppose a sequential cascade of events leading to indirect and stable events, not only related to differential expression of transcription factors but also to alteration in the epigenetic markers and ultimately in MCF-7R cell fate.

#### 2.2.4. Differentially Methylated Non-Coding RNAs

Non-coding RNAs account for 30% of the demethylated genes following curcumin treatment of MCF-7R cells. Table 3 highlights the most significantly differentially methylated ncRNA genes, selected based on methylation differences, the number of CpG sites in the DMRs, or the number of tiles per gene. These genes are methylated when cells acquire the MDR phenotype and become demethylated upon curcumin treatment.

Non-coding RNAs, both long non-coding RNAs (lncRNAs) and microRNAs (miRNAs), are involved in the regulation of gene expression (transcriptionally or post-transcriptionally), chromatin structure, and various cellular processes. In particular, curcumin reverts the miR-663, MIR34AHG, and HALGR/HALGROS methylation acquired in MCF-7R.

MiR-663 directly targets the eukaryotic translation elongation factor A2 (*eEF1A2*), which is normally utilized by neuronal, cardiac, and muscle cells to promote the elongation process during translation. *EF1A2* is considered a proto-oncogene overexpressed in MCF-7 cells. Downregulation of *eEF1A2* by miR-663 has been shown to inhibit the proliferation of MCF-7 cells [33]. Additionally, miR-663 also targets *TGFβ1*, where its inhibition suppresses tumor growth and epithelial–mesenchymal transition (EMT) [34,35]. Moreover, miR-663 activates the canonical Wnt signaling through the APC suppression [36].

Based on this evidence, this miRNA is increasingly recognized as a tumor suppressor, and it is downregulated in human gastric cancer cells, leading to mitotic catastrophe and growth arrest [37]. However, in nasopharyngeal carcinoma cells, miR-663A may act as an oncogene by promoting cell growth and tumorigenesis, targeting the cyclin-dependent kinase inhibitor p21WAF1/CIP1 [38].

*MIR34AHG* is the precursor gene for miR-34a. The microRNA miR-34a directly suppresses several transcripts, like the *NOTCH1*, *CDK6*, and *MET* transcription factors, and therefore functions as a tumor suppressor and is frequently downregulated across multiple cancer types, including breast cancer. The downregulation of the miR-34 gene by DNA methylation has been associated with an elevated risk of metastasis and advanced stages of ovarian, breast, and small-cell lung cancer [39,40,41]. Additionally, the downregulation of the miR-34 gene has also been observed in MCF7R cells, while a treatment with the demethylating agent 5-azacytidine results in an increased miR-34 level [42]. The same region, hypermethylated in MCF-7R and hypomethylated in MCF-7RC, in the opposite direction produces *LNCTAM34A* (also known as Lnc34a), a long non-coding transcriptional activator of miR34a [43].

In general, miRNAs influence drug-induced apoptosis by targeting apoptosis-related proteins or drug resistance pathways. In particular, several miRNAs, such as miR-34a, miR-7-5p, and miR-325-3p, have also been reported to play a role in anticancer drug resistance by influencing ABC transporters in some tumor models such as colon cancer, glioblastoma, and hepatocellular carcinoma. Also, in a chemoresistant gastric carcinoma model, miR-633 inhibited doxorubicin (DOX)/cisplatin (CDDP)-induced apoptosis cells by downregulating FADD via directly targeting its 3′UTR [44].

*HALGR* (also known as *HOXD-AS*1 and *Mdgt*) is located between *HOXD*1 and *HOXD3* and encodes a lncRNA reported to play important roles in gut development and processes associated with various cancers. HALGR epigenetically represses transcription (p57) through recruiting the enhancer of zeste homolog 2 (EZH2) to the promoter. In cervical cancer, this promotes cell proliferation, colony formation capacity, and chemoresistance to cisplatin [45]. However, to date, it has not been reported in breast cancer [46].

### 2.3. Effects of Curcumin on ABCB1 Transcription Regulators

#### 2.3.1. DNA Methylation: Several TFs Are Differentially Methylated

Although RRBS results highlight that at 24 h, curcumin has no effect on *ABCB1* DNA methylation, the observed alteration in protein and mRNA expression suggests that other mechanisms are involved. Therefore, we evaluated the methylation pattern of the known regulators of *ABCB1* expression.

Regarding the Wnt pathway, several TCF/LEF sites lying between −1813 and −261 within the *ABCB1* promoter were previously found [47]. The methylation difference was inspected for all members of this family; however, after exposure to curcumin, no significant variations in genes of the TCF/LEF family were observed. Differently, the *TCF15* (linked to MAPK Signaling and Integrin-linked kinase Signaling) promoter was strongly demethylated. Intriguingly, as described in a previous section, several regulatory regions in the *WNT* gene family result demethylated after the treatment with curcumin and are methylated in the acquisition of the MDR phenotype, like *WNT3* and *WNT9B*.

The AP-1 complex is also a known regulator of *ABCB1* transcription. c-fos binding elements are located on the promoter of *ABCB1* and can be involved in transcriptional activation or repression [48]. However, following curcumin treatment, the promoter methylation of *FOS* does not show significant changes. Similarly, curcumin does not affect *ETS1, ESR2, EGR1*, or *WT1* (encoding the Wilms’ tumor suppressor protein) gene methylation, which are known as transcriptional regulators of *ABCB1* [49,50,51].

Differently, other transcription factors can bind the regulatory region of *ABCB1* (ReMap ChIP-seq data [52]) and some of them are affected in their DNA methylation levels. *HOXB13*, encoding a homeobox transcription factor that regulates the *ABCB1* gene at least in prostate cancer, is demethylated in the promoter after treatment with curcumin. Moreover, *CBX4* (hypermethylated in MCF-7R), *RING1* (ring finger protein 1), *ZNF232/ZSCAN11* (Zinc Finger and SCAN Domain-Containing Protein 11), *OSR2* (odd-skipped related transcription factor 2), and *TFAP2A* (transcription factor AP-2 alpha) are all demethylated.

#### 2.3.2. Expression Analysis: *Egr1* and *Ezh2* Transcripts Change Their Levels in MCF-7R Cells Treated with Curcumin

In order to assess if some of the major regulators of *ABCB1* expression, even if not differentially methylated in their genes, such as NF-κB, could be altered in their expression, we performed RT-qPCR experiments.

**Figure 10:**
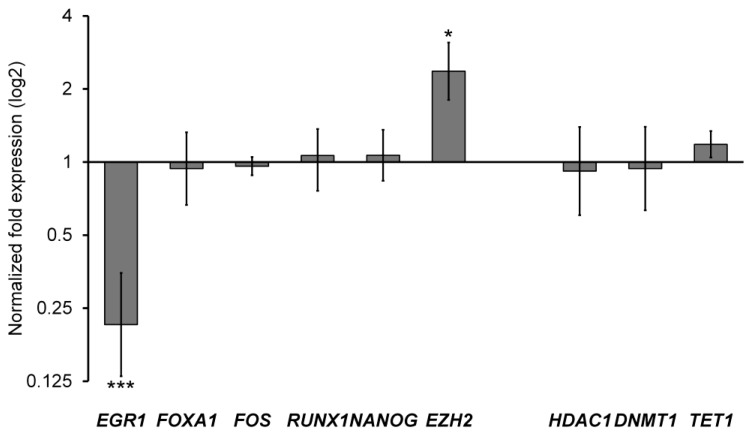
Results of RT-qPCR experiments performed on *ABCB1* regulators and chromatin regulators. ***: *p* value < 0.0005, *: *p* value < 0.05.

Regarding the expression of genes related to the regulation of the epigenetic landscape, we did not observe any significant difference in the expression of *HDAC*, *DNMT1*, and *TET* (Figure 10).

The results show that *FOS, RUNX1*, and *NANOG* maintain the same transcript levels; differently, *EGR1* and *EZH2* change their expression significantly (Figure 10).

EGR1 is known to act as a transcriptional activator of *ABCB*1, via direct binding of GC elements on the proximal promoter [53]. EZH2 is the methyltransferase subunit of the Polycomb Repressive Complex 2 (PRC2), having the H3K27me3 activity known to label poised promoters/enhancers. Remap ChIP-seq data showed that *ABCB1* is a target of PRC2 in different cell types and tissues; however, a direct relationship between the EZH2 expression and *ABCB1* level was not always defined, mainly due to the different cellular context [54].

Herein we show that at least two transcriptional regulators (EGR1 and EZH2) undergo changes in their mRNA levels, thus presumably altering the complex combinatorial regulation of the gene. Thus, we can hypothesize that curcumin acts on various transcriptional factors of *ABCB1*, besides NF-κB, through different levels and mechanisms of regulation. Reasonably, curcumin, likely acting on various transcriptional factors that regulate *ABCB1*, could disrupt the coordination of signals (difficulties in finding a new balance through a difficult combinatory control) that can require a long and continuous exposure time for an evident effect at the transcriptional level.

## 3. Materials and Methods

### 3.1. Cell Lines

The MCF-7 cell line was obtained from ATCC (HTB-22™, Manassas, VA, USA). The multidrug resistance (MDR) cell line MCF-7R was derived treating the wild-type cells with gradually increasing concentrations of doxorubicin. The IC_50_ value of doxorubicin in MCF-7R is approximately 75 times higher than the original IC_50_. MCF-7R cells lack ERα expression, are estrogen-insensitive, overexpress P-gp, and are characterized by the overactivation of NF-κB. Cell lines were cultured in Dulbecco’s Modified Eagle Medium (DMEM) (HyClone Europe Ltd., Cramlington, UK), supplemented with 10% heat-inactivated fetal calf serum, 2 mM L-glutamine, 100 units/mL penicillin, and 100 μg/mL streptomycin (all reagents were from HyClone Europe Ltd., Cramlington, UK). All cell lines were cultured in a humidified atmosphere of 5% CO_2_ at 37 °C. The cultures were routinely tested for mycoplasma infection. Cells with a narrow range of passage numbers were used for all experiments.

For the cell viability assay (MTS, Promega Corporation Madison, WI, USA), cell lines were seeded at 5 × 10^3^ cells/well in 96-well plates and after 24 h, curcumin at several concentrations was added. After 24 or 72 h of treatment, 16 μL of MTS dye was added and its bioreduction was evaluated by measuring the absorbance of each well at 490 nm using a microplate reader (iMark Microplate Reader; Bio-Rad Laboratories Inc., Hercules, CA, USA).

To evaluate the effects of curcumin, the cells were cultured in 24-well plates at a density of 1 × 10^5^ cells per well. After 24 h, the cells were treated with curcumin (30 µM) per 24 h of incubation.

### 3.2. Western Blotting

MCF-7R cell lysates were obtained using RIPA lysis buffer (Santa Cruz Biotechnology Inc., Dallas, TX, USA). First, 25 μg of proteins were subjected to an electrophoretic run on 10% SDS-PAGE acrylamide gel and then transferred to Hybond-P membrane (GE Healthcare Europe GmbH, Freiburg, Germany). Then, the filters were incubated with GAPDH primary antibodies (0411; sc-47724 mouse 1:10,000); P-gp (cat. n.13,854, Invitrogen; mouse 1:200). Hybridization was visualized using an enhanced chemiluminescence detection kit (SuperSignal West Femto Maximum Sensitivity Substrate, Thermo Scientific Life Technologies Italia, Monza, Italy) and the Versa DOC imaging system (BioRad Laboratories, Milan, Italy). Immunoblots were quantified by densitometry and the results were expressed as arbitrary units (protein/GADPH).

### 3.3. NF-κB Activation

The DNA-binding capacity of NF-κB (p65 subunit) was measured in the nuclear extracts of MCF-7R cells after treatment with curcumin, 30 µM (Sigma Aldrich srl, Milan, Italy) for 24 h by the TransAM™ NF-κB and Nuclear Extract™ Kits (Active Motif, Carlsbad, CA, USA) according to the manufacturer’s instructions. Briefly, the determination is based on the ability of activated NF-κB contained in nuclear extracts to specifically bind an oligonucleotide containing the NF-κB consensus binding site (5′-GGGACTTTCC-3′) immobilized at the bottom of a 96-well plate. Using an antibody directed against an epitope on p65, accessible only when NF-κB is bound to its target DNA, NF-κB bound to the oligonucleotide is detected. The addition of a horseradish peroxidase–conjugated secondary antibody provides a sensitive colorimetric readout that is quantified by densitometric analyses. The specificity of the test is confirmed by simultaneous incubations in the presence of an excess of non-immobilized consensus oligonucleotide, as a competitor, or of a mutated consensus oligonucleotide. The results were expressed as arbitrary units: one unit represents the DNA binding capacity shown by 2.5 μg of whole cell extract from Jurkat cells stimulated with 12-O-Tetradecanoylphorbol-13-acetate (TPA) + calcium ionophore (CI)/μg protein of nuclear extracts.

### 3.4. Reduced Representation Bisulfite Sequencing (RRBS) and Differential Methylation Analysis

Genomic DNA was extracted using the Genomic-tips Qiagen from MCF-7, MCF-7R, and MCF-7R treated with curcumin (MCF-7RC); two biological replicates for each sample. gDNA was quantified with spectrophotometer and fluorimetric method (Qubit™ dsDNA HS Assay Kit, Invitrogen Ltd., Paisley, UK) and integrity was evaluated on 1% agarose gel. RRBS libraries were generated following the Premium RRBS kit v2 (Diagenode, Liège, Belgium) protocol, starting from 100ng genomic DNA. Libraries were quantified with the Qubit™ dsDNA HS Assay Kit and analyzed with the High Sensitivity D1000 ScreenTape Assay kit on the Agilent TapeStation 4200 instrument (Agilent, Santa Clara, CA, USA). Libraries were sequenced on the Illumina NovaSeq platform using 150-bp paired-end reads. Raw fastq reads were initially quality-tested using FastQC (“https://www.bioinformatics.babraham.ac.uk/projects/fastqc/ (accessed on 25 October 2024)”). Subsequently they were aligned against the reference human genome (GRCh38/hg38) using the bisulfite-specific short read aligner bsmap v 2.90 [55]. The restriction enzyme digestion site parameter was set to “C-CGG” for MspI digestion. Following alignment, bam files were sorted and indexed using samtools [56]. The methylation ratio on individual samples was calculated using the bsmap v 2.90 methratio script.

The data quality assessment was performed using the R (version 4.3.2) package methylKit (version 1.28) and bimodal CpG Methylation % profiles were obtained. The general coverage statistics were checked, and the samples were filtered based on coverage (minimum coverage >3 or >10). The mean coverage obtained on these CpG sites ranged from 21 to 25 between six methylomes.

Differential analysis was performed on both individual CpGs (DMCs) and 200-bp tiles (DMRs—1000 bp tiles were used just for visualization in the genome browser) using the *methylKit* R package. Regarding the DMC method analysis (individual CpG method), DMC were selected considering coverage cutoff = 10, q value cutoff = 0.01, |diff.meth| cutoff = 25. Tile-based method analysis was performed using default parameters: q-value < 0.01 and minimum coverage in the tile equal to 10. Tiles with a |diff.meth| greater than 25% were considered significant [57]. All data were filtered and assembled in a dataframe with a row for each region 200bp long, considering as methylation difference the average of data obtained from the two analyses (individual CpGs and tiles). Only 200 bp DMRs with a |diff.meth mean| >= 25% and a number of DMCs > 3 were considered significant.

Using the R package *genomation* (version 1.34), the regions corresponding to the selected tiles were then annotated to assess whether they corresponded to genes or specific regulation elements (cCRE, CGI tracks). To obtain the latest annotation version, the data were downloaded using the Table Browser tool from the UCSC (University of California Santa Cruz) Genome Browser, or directly from ENCODE portal (“https://genome-euro.ucsc.edu/cgi-bin/hgGateway?redirect=manual&source=genome.ucsc.edu (accessed 15 November 2024)”). The region that extends from −1000 to +1000 bp with respect to the transcription start site (TSS) was considered as “promoter”.

For data management, genomic ranges (version 1.54.1) and dplyr (version 1.1.4) R packages were also used.

KEGG and Gene Ontology analysis were performed using the ShinyGO (version 0.81) tool (“http://bioinformatics.sdstate.edu/go/ (accessed 25 October 2024)”) [58].

### 3.5. Reverse Transctiption and Quantitative PCR

Gene expression was evaluated on the MCF-7R and MCF-7RC cell lines. Total cellular RNA was isolated using TRIzol reagent (Invitrogen Life Technologies, Carlsbad, CA, USA) according to the manufacturer’s instructions. RNA quantity and quality were inspected by Biofotometer and by gel electrophoresis. For each sample, the same amount of total RNA (100 ng) was reverse-transcribed using a High-Capacity cDNA Reverse Transcription Kit (Applied Biosystems, Foster City, CA, USA) following the protocol provided by the manufacturer. cDNA quality was tested by endpoint PCR using GAPDH primers, and then qPCR reactions were performed using BrightGreen 2X qPCR MasterMix (Applied Biological Materials Inc., Richmond, BC V6V 2J5 Canada) in triplicates. The primer list and sequences are shown in Table 4.

The running of the samples and data collection were performed on a StepOne AB Real Time PCR system (Applied Biosystems Life Technologies Inc., Foster City, CA, USA). *GAPDH* and *PCBP1* transcripts were used as an internal standard.

### 3.6. Statistical Analysis

The results obtained are reported as mean ± standard error (SE). Statistical analysis was performed by analysis of variance (one-way ANOVA) followed by Tukey’s test. STATISTICS ver. 10 (StatSoft Inc. 2011, Tibco Software, Stanford Research Park Palo Alto, CA, USA), Tibco Software, Stanford Research Park Palo Alto, CA, USA) was used as analysis software.

## 4. Conclusions

In this work, we evaluated the effects of curcumin on MCF-7R multidrug-resistant cells. In particular, we observed the expression of P-gp and some of its regulators. Moreover, we inspected the DNA methylation landscape after curcumin exposure showing specific changes in the methylation rate of defined loci. Curcumin causes the demethylation of ribosomal genes (*45S* and *5S*) and genes related to ribosomal maturation and tRNAs, likely impairing ribosome assembly and protein synthesis. Moreover, curcumin exposure affects the methylation of transcription factors and chromatin writers, suggesting long-term cascades of events independently from the direct DNMT inhibition exerted by curcumin. All these effects contribute to reverting the MDR phenotype acquired by the MCF-7 cell after doxorubicin treatments. Further studies are ongoing to deepen the knowledge about the molecular mechanisms of MCF-7 multidrug resistance related to DNA methylation.

## Figures and Tables

**Table 1:** DNA binding capacity of NF-κB (p65 subunit) in nuclear extracts of MCF-7R. Cells were treated for 24 h with curcumin (30 µM = IC_50_ value at 72 h).

Treatments	Arbitrary Units/μg of Cell Nuclear Extract Protein
Control	0.7 ± 0.001
Curcumin 30 µM	0.06 ± 0.002 *

Results are expressed as arbitrary units/μg of cell nuclear extract protein. The results are expressed as the mean ± standard error of two different experiments in triplicate. Differences when treatment is compared to control: * *p* < 0.001 (Tukey’s test).

**Table 2:** Chromatin and transcription regulators.

Symbol	diff.meth Mean (R-S)	diff.meth Mean (RC-R)	Prom	CCRE	CGI (Shore)	Description
*H1-0*	66	−31	0	1	1 (1)	H1.0 linker histone
*H3C8*	37	−28	1	1	1 (1)	H3 clustered histone 8
*MEN1*	53	−38	1	1	1 (0)	menin 1 (HMT complex)
*CBX2, CBX8*	63	−25	0	1	1 (0)	chromobox 2/8 (PRC1 complex)
*CBX4*	61	−28	0	1	1 (0)	chromobox 4 (PRC1 complex)
*RING1*	47	−27	0	1	0 (1)	ring finger protein 1 (PRC1 complex)
*MBD3L1*	58	−34	1	1	1 (0)	methyl-CpG binding domain protein 3 like 1 (Does not bind methylated DNA)
*NOC2L*	63	−31	1	1	1 (0)	NOC2 like nucleolar associated transcriptional repressor (INHAT)
*HOXB13*	46	−30	1	1	1 (1)	homeobox B13
*HOXD1*	59	−37	1	1	1 (1)	homeobox D1
*DLX4*	36	−28	0	1	1 (1)	distal-less homeobox 4
*MSX1*	72	−27	0	1	1 (1)	msh homeobox 1
*NKX2-5*	35	−27	0	1	1 (0)	NK2 homeobox 5
*ZNF503/Nlz2*	46	−26	1	1	1 (0)	zinc finger protein 503 (transcriptional repressor)
*TRIM27*	82	−27	0	1	0 (0)	tripartite motif containing 27 (transcriptional repressor)
*MLXIPL*	59	−30	1	0	1 (0)	MLX interacting protein like (TF of the Myc/Max/Mad superfamily)
*TOX2*	44	−33	1	1	1 (0)	TOX high mobility group box family member 2

HMT: MLL/SET1 histone methyltransferase; INHAT: HDAC-independent inhibitor of histone acetyltransferase.

**Table 3:** RRBS analysis results: the most significant DMRs in non-coding genes. Gene symbol, methylation difference (at q-value < 0.01) between MCF-7R and MCF-7, methylation difference (at q-value < 0.01) between MCF-7R treated with curcumin and MCF-7R, functional genomics characteristics (Promoter, CCRE, CGI) are shown. The last column shows the gene description. For the complete results, see Table S1.

Symbol	diff.meth Mean (R-S)	diff.meth Mean (RC-R)	Prom	CCRE	CGI (Shore)	Description
*HAGLR/ HALGROS*	53	−29	1	1	1 (0)	HOXD antisense growth-associated long non-coding RNA/HAGLR opposite strand lncRNA
*FAM182B*	72	−51	1	1	0 (0)	family with sequence similarity 182 member B, lncRNA
*MIR663A*	35	−30	1	1	1 (0)	microRNA 663a
*MIR34AHG*	69	−30	1	1	1 (0)	microRNA 34a host gene
*MIR3648-1*	29	−36	1	0	0 (0)	microRNA 3648-1
*MIR6724-2*	36	−28	1	0	1 (0)	microRNA 6724-2
*LINC01623*	82	−27	0	1	0 (0)	long intergenic non-protein coding RNA 1623

**Table 4:** List of primer sequences utilized in RT-qPCRs.

Target	FORWARD	REVERSE
*DNMT1*	GTCATGAACTCCAAGACCCACC	AGCGCCTCATAACTCTCAAAGC
*HDAC1*	TATCGCCCTCACAAAGCCAATG	AGTTTCACAGCACTTGCCACAG
*TET1*	AAAGAAGAGGGCTGCGATGATG	ACGGTCTCAGTGTTACTCCCTAAG
*EZH2*	TGTTTCCAGATAAGGGCACAGC	ACTCCTTTGCTCCCTCCAAATG
*FOXA1*	CACAGGGCTGGATGGTTGTATTG	GAGTAGGCCTCCTGCGTGTC
*FOS*	CCAAGCGGAGACAGACCAACTA	CATTGAGGAGAGGCAGGGTGAA
*RUNX1*	CGGTCGAAGTGGAAGAGGGAAA	ATCTCCAGGGTGCTGTGTCTTC
*NANOG*	CAGCTACAAACAGGTGAAGACC	AGTCACTGGCAGGAGAATTTGG
*EGR1*	CCGCAGAGTCTTTTCCTGACATC	TAAATGGGACTGCTGTCGTTGG
*ABCB1*	ACTAGAAGGTTCTGGGAAGATCGC	TGTGGGCTGCTGATATTTTGGC
*GAPDH*	GACAGTCAGCCGCATCTTCT	TTAAAAGCAGCCCTGGTGAC
*PCBP1*	TGATCATCGACAAGCTGGAG	TCTTTGATCTTACACCCGCC

## Data Availability

The original contributions presented in this study are included in the article/Supplementary Material. Further inquiries can be directed to the corresponding author.

## Supplementary Materials

The following supporting information can be downloaded at: https://www.mdpi.com/article/10.3390/ijms252413416/s1.
