# Supplementary material for: Epigenetic and Cellular Reprogramming of Doxorubicin-Resistant MCF-7 Cells Treated with Curcumin
Source: Int J Mol Sci. 2024 Dec 14;25(24):13416. doi: 10.3390/ijms252413416 (PMC11679585; doi:10.3390/ijms252413416)
Supplement: Supplementary file 1 [file ijms-25-13416-s001.zip › Supplementary material S1.pdf]

Supplementary material S1

Article:

Epigenetic and Cellular Reprogramming of Doxorubicin-Resistant MCF-7 Cells Treated with Curcumin

Paola Poma, Salvatrice Rigogliuso, Manuela Labbozzetta, Aldo Nicosia, Salvatore Costa, Maria Antonietta Ragusa and Monica Notarbartolo

International Journal of Molecular Sciences

Corresponding author: Maria A. Ragusa, maria.ragusa@unipa.it

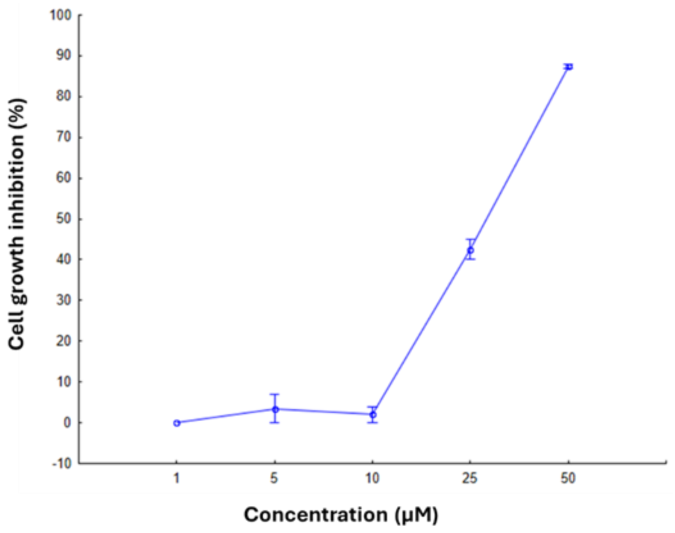

Figure S1. MTS analysis of Curcumin treatment on the MCF-7R cell line: cytotoxic effects observed after 72 h of exposure.

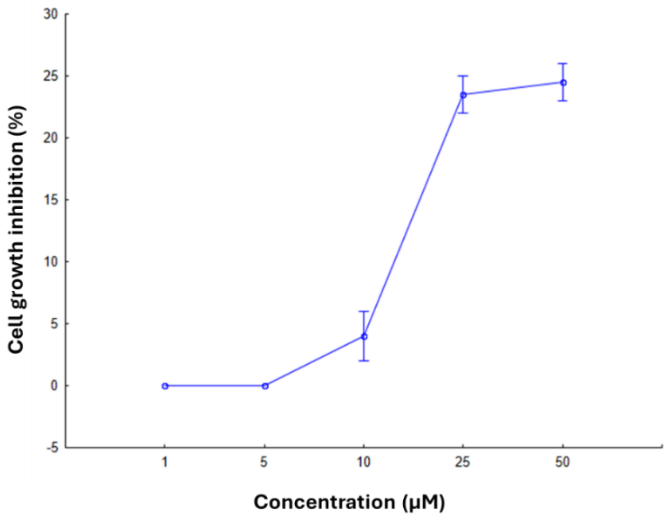

Figure S2. MTS analysis of Curcumin treatment on the Human Mammary Epithelial Cells, (HMEpiC, ref. P10891) cell line: cytotoxic effects observed after 24 h of exposure.
